# Impressions on European physiology education and research—A quick view from the Portuguese experience

**DOI:** 10.14814/phy2.70148

**Published:** 2024-12-27

**Authors:** Luís Monteiro Rodrigues

**Affiliations:** ^1^ Universidade Lusófona Lisbon Portugal; ^2^ Portuguese Society of Physiology (SPF) Lisbon Portugal

## Abstract

The Bologna Process and the European Higher Education Area (EHEA) are celebrating 25 years of free mobility and career opportunities. Despite its progress, disparities exist in the effectiveness of this initiative across EHEA countries. Physiology is a mandatory component of health science curricula but lacks standardized educational benchmarks, leading to limited societal awareness and professional opportunities. In contrast, there is a different dynamic in the USA, where physiology is a profession with growing opportunities. Within Europe, national scientific societies are actively promoting physiology education and research but societal awareness and physiology related knowledge and opportunities are still limited. A recent study on the Portuguese reality revealed that while physiology is taught at many institutions, there is significant variability in hands‐on training between universities and technical colleges. Notably, only a small percentage of faculty have published in physiology journals, suggesting a diluted identity for the discipline. Challenges in harmonization and training reflect a need for stronger collaboration among scientific organizations to improve physiology teaching and research across Europe. By addressing these issues, the future of physiology can be strengthened, ensuring better recognition and professional development for physiologists in the EHEA.

## Context

The Bologna Process and the European Higher Education Area (EHEA) are currently celebrating 25 years. This transforming initiative is ultimately responsible for free circulation of students within the European space (and beyond) during their education (European Commission/EACEA/Eurydice, 2020; European Education and Culture Executive Agency, 2024) and extends to professionals, as the automatic recognition of diplomas has widened the work market and (career) opportunities as never before (European Commission, 2020). However, over the balance of this period, we in the field confirmed what was anticipated—that this process has progressed and continues to progress with different rhythms and efficacy within the EHEA, no matter the common goals and dramatic changes involving harmonization and mobility.

Physiology is currently a mandatory discipline in all courses of human health sciences across Europe, clearly regarded as fundamental. However, European Physiology standards for education do not exist. Physiology as a professional career also does not exist in most countries within the EHEA; consequently, societal awareness regarding its applicability and its opportunities is very limited. A very different context exists within the USA, mostly based on structured STEM (educational) systems, and yet physiologists from both worlds seem to share the same concerns and views regarding the Future of Physiology (Gregorio, 2019; IUPS, 2016; Rodrigues et al., 2023). National scientific societies are widely present in Europe and show, at least in some cases, remarkable dynamics (https://www.physoc.org/) promoting education and research, actively mobilizing interested communities around their proposals. Aggregation organizations and initiatives such as FEPS (https://www.FEPS.org) or Europhysiology (https://www.physoc.org/events/europhysiology‐2022/) are increasingly visible and active on these global stages. Various peer‐reviewed journals specify Physiology in their titles, and in many cases are linked to those societies. A few are long‐established, but new editorial initiatives continue to emerge in the area (e.g. Elsevier launched new journals to advance nutrition and precision medicine).

**So, what is needed to promote physiological awareness in the general population?**

**To improve the visibility of Physiology especially for young people?**

**What can we do to gather European Physiology and physiologists and create a European identity?**

Our own experience in the SPF—the Portuguese Physiological Society—has shown that it is important to understand the current reality of Physiology teaching and research within the EHEA to create an informed community. We recently initiated this study in Portugal (Rodrigues et al., 2024) and confirmed that Physiology is a mandatory discipline in all human health‐related courses at universities and technical colleges, the two main structures in the system. Once exclusive at medical schools, Physiology is now present in Medicine, naturally, but in many other university courses such as Pharmacy (Integrated Masters), Physical Education and Sports, Nutrition, Biology Biochemistry and Biomedical Engineering (1st cycle bachelors) as well. Nursing, Health Technologies (including physiotherapy) and Nonconventional Therapies (1st cycle bachelors) administered in technical colleges involve the biggest number of training hours and credits, especially in the 1st year. Nevertheless, our analysis has shown that the hands‐on component greatly differs between universities and technical colleges, which we considered to be related to differences in education and training in Physiology among the faculty (Rodrigues et al., 2024).

We also looked to the bibliometric profile of the faculty member heading the Physiology discipline, where available, between 2017 and 2022 by using their respective ORCID identifier. From the original sample, only ca. 25% of the physiology units contained this information, and from these only 14.6% showed a publication track with indexed publications or conference papers and abstracts in that period. The large majority (2/3) belonged to universities, 50% of which belonged to Medicine programs. Almost 40% of those responsible faculty members produced 0–5 indexed publications in this period (according to Web of Science and PubMed) while only 15.6% had more than 30 indexed publications in the same period. Again, the highest outputs were clearly linked to Medical School staff, although we could find higher impacts measured by their H‐index in other areas. A significantly higher scientific output (*p* = 0.011) was detected for the universities compared to the technical colleges (Rodrigues et al., 2024). Finally, we also looked at these authors' journal choices, considering the physiological denomination included in the journal title. As shown in Table 1, the most chosen journal thematic area was “Medicine (general or internal)” representing 37.6% of publications, followed by Biochemistry, Biology, and Genetics journals. Only five authors published in Physiology journals (4.6%)—that is, journals with the words “physiology/physiological” in their denomination (Table 1). From this data, of course, we cannot determine if the journals of publication were the authors' preferred venue or no. Whether by choice or necessity, this reality dilutes the identify of Physiology.

**TABLE 1:** Most chosen journal thematic area within the referred study sample. See text for details of the study sample and procedure.

Journal thematic area	% of publishing authors
Medicine (general, internal)	37.6%
Biochemistry, Genetics, Molecular and Hereditary Biology	22.1%
Physiology	4.6%
Other	35.7%

Although we can identify limitations in this study, we believe this profile is likely very similar to what we might find in other EHEA countries, especially in central and southern Europe. Nevertheless, a few points might be highlighted:

1. The hands‐on component of Physiology differs greatly between universities and technical colleges. The dramatic expansion of the discipline from exclusively medical to other courses, especially in technical colleges, was not met by an adequate training of new physiologists. As a result, Physiology seems to be taught by other professionals who have no previous training in this field, which can be expected to directly impact program contents and competencies.
2. Related, faculty with relevant production are primarily affiliated with reference university centers or research units, and significant differences exist when comparing faculty research production between universities and technical colleges. We believe that teaching supported by research is essential in a discipline such as Physiology. Based on demonstration Physiology teaching demands a different level of training to utilize special teaching‐learning communication tools.

Noteworthy, among the journal thematic areas detected in our sample, less than 5% of the publications were within Physiology journals. Again, the authors´ options might be by choice or necessity. It is clear that harmonization processes face multiple difficulties and run at different velocities in such a complex environment. Therefore, to ensure a future for Physiology in Europe, scientific organizations, both local (national) and global (European), must assume a leading role in preserving the core principles of this knowledge area and ensuring its progress. Closely articulated, these organizations might be able to (a) better understand the reality of Physiology (teaching, learning, and research) in their own countries and within EHEA to (b) mobilize and prepare physiologists for future challenges based on the objective review of current threats and opportunities, and to (c) set the basis to establish a strong physiologist profile in terms of knowledge, practical experience, and competences. We believe these efforts will definitively mark a new era for Physiology and physiologists in Europe.

## AUTHOR'S DISCLAIMER

The views expressed in this editorial represent my own opinions and do not necessarily represent the views or opinions of the SPF or Universidade Lusófona.
